# Supplementary material for: Kinetic Extraction of Fucoxanthin from Undaria pinnatifida Using Ethanol as a Solvent
Source: Mar Drugs. 2023 Jul 21;21(7):414. doi: 10.3390/md21070414 (PMC10381705; doi:10.3390/md21070414)
Supplement: Supplementary file 1 [file marinedrugs-21-00414-s001.zip › marinedrugs-2478921-supplementary.pdf]

Supplementary Material

Table S1. *MAE*. *RMSE* and *RMSE-MAE* values for each response (*Y*) at different *T* and *S*.

VARIABLES		Y_1 (µg Fx/g AS)			Y_2 (mg E/g AS)			Y_3 (mg Fx/g AS)		
S (%)	***T*** (ºC)	*MAE*	*RMSE*	*RMSE-MAE*	*MAE*	*RMSE*	*RMSE-MAE*	*MAE*	*RMSE*	*RMSE-MAE*
	5	89.5	122	32.5	15.3	19.5	4.3	0.3	0.4	0.1
	25	153.6	194.4	40.8	10.5	14.9	4.4	0.6	0.9	0.3
**50**	45	50.8	59.5	8.8	16.4	21.7	5.3	0.4	0.5	0.1
	65	228	262.2	34.2	8.5	13.1	4.6	0.6	0.7	0.1
	85	173.4	289	115.6	6.5	15.9	9.3	0.6	1.4	0.8
	5	390.8	445.1	54.3	15.1	17.6	2.4	1.4	1.6	0.2
	25	179.6	206.7	27.1	10.7	14.5	3.8	0.5	0.5	0.1
**60**	45	215.9	257.3	41.4	14.3	20.6	6.3	0.6	0.7	0.1
	65	391.2	501.4	110.2	9.5	12.5	3.0	0.3	0.6	0.3
	85	320.1	411	90.9	6.9	9.4	2.5	2.1	2.6	0.5
	5	935.2	1039.2	104.0	17.7	22.0	4.3	1.9	2.2	0.3
	25	157.9	217.2	59.3	4.6	6.5	1.9	0.3	0.4	0.1
**70**	45	166.4	198.2	31.8	19.5	27.4	7.9	0.5	0.6	0.1
	65	302.3	418.8	116.5	9.0	11.2	2.2	0.7	1.0	0.3
	85	292.2	384.6	92.4	9.5	13.9	4.4	2.1	2.7	0.7
	5	726	838.2	112.2	18.0	23.9	5.9	2.3	2.6	0.4
	25	274.9	368.5	93.6	6.9	9.4	2.5	0.9	1.1	0.2
**80**	45	283.5	323.1	39.6	10.1	12.4	2.3	0.7	0.8	0.2
	65	135.8	189.6	53.8	17.7	23.8	6.1	0.9	1.2	0.3
	85	361.9	482.7	120.9	11.2	16.3	5.2	2.4	3.0	0.7
	5	427.4	592.9	165.4	18.2	22.4	4.3	2.4	2.9	0.4
	25	405.3	469.6	64.3	23.2	27.4	4.2	1.5	1.9	0.4
**90**	45	401.8	461.3	59.6	45.5	54.4	8.9	0.9	1.2	0.2
	65	267.9	300.2	32.3	20.7	23.9	3.2	0.8	1.1	0.3
	85	311.1	392.5	81.4	18.5	24.0	5.5	2.1	3.1	0.9
	5	511.9	748.2	236.4	10.2	12.4	2.2	5.7	7.1	1.3
	25	220.3	295.2	75.0	14.6	17.5	2.9	5.5	7.7	2.1
**100**	45	384	443.1	59.1	7.7	10.0	2.2	3.0	3.7	0.7
	65	242.3	326.1	83.8	10.0	12.7	2.7	4.6	5.9	1.3
	85	344.2	429.7	85.5	12.1	17.5	5.4	4.7	6.7	2.0

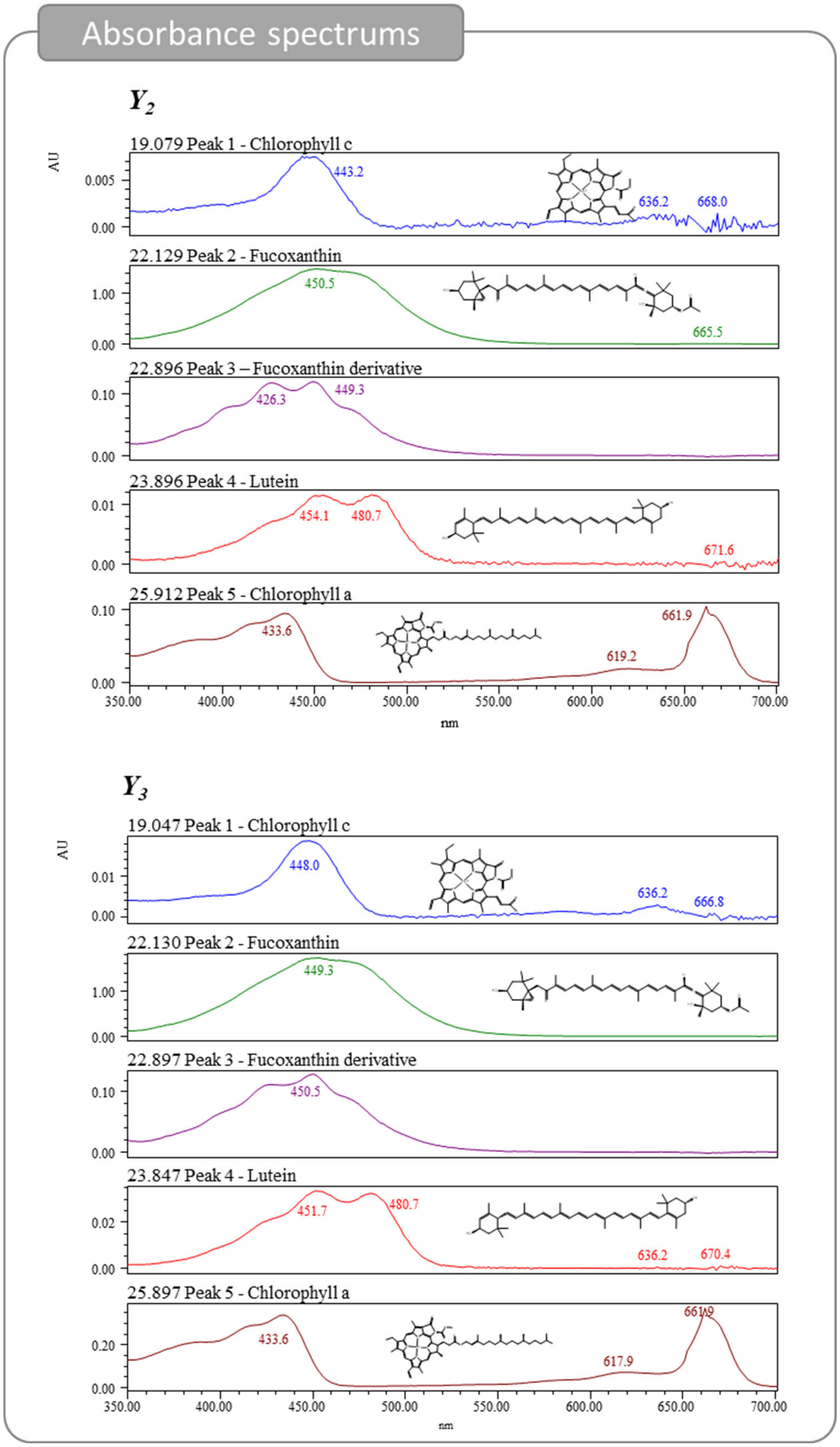

Figure S1. Absorption spectrums for the five identified pigments found in Y_2 and Y_3 using HPLC-DAD.
